# Control of circadian rhythm on cortical excitability and synaptic plasticity

**DOI:** 10.3389/fncir.2023.1099598

**Published:** 2023-03-30

**Authors:** Claudia Lodovichi, Gian Michele Ratto

**Affiliations:** ^1^Institute of Neuroscience, Consiglio Nazionale delle Ricerche (CNR), Padova, Italy; ^2^Veneto Institute of Molecular Medicine (VIMM), Padova, Italy; ^3^Padova Neuroscience Center, Universitá degli Studi di Padova, Padova, Italy; ^4^National Enterprise for NanoScience and NanoTechnology (NEST), Istituto Nanoscienze, Consiglio Nazionale delle Ricerche (CNR) and Scuola Normale Superiore, Pisa, Italy

**Keywords:** circadian rhythm, mTOR, LTP, memory and learning, neuronal excitability, chloride homeostasis

## Abstract

Living organisms navigate through a cyclic world: activity, feeding, social interactions are all organized along the periodic succession of night and day. At the cellular level, periodic activity is controlled by the molecular machinery driving the circadian regulation of cellular homeostasis. This mechanism adapts cell function to the external environment and its crucial importance is underlined by its robustness and redundancy. The cell autonomous clock regulates cell function by the circadian modulation of mTOR, a master controller of protein synthesis. Importantly, mTOR integrates the circadian modulation with synaptic activity and extracellular signals through a complex signaling network that includes the RAS-ERK pathway. The relationship between mTOR and the circadian clock is bidirectional, since mTOR can feedback on the cellular clock to shift the cycle to maintain the alignment with the environmental conditions. The mTOR and ERK pathways are crucial determinants of synaptic plasticity and function and thus it is not surprising that alterations of the circadian clock cause defective responses to environmental challenges, as witnessed by the bi-directional relationship between brain disorders and impaired circadian regulation. In physiological conditions, the feedback between the intrinsic clock and the mTOR pathway suggests that also synaptic plasticity should undergo circadian regulation.

## Introduction

The alternation of light and darkness dictates adjustments to all living organisms in the form of alternate periods of rest and activity. This pattern is present at all complexity scales from humans all the way down to unicellular organisms. In eukaryotic cells time keeping is primarily due to a cell autonomous machinery that relies on transcription as well as on post-transcriptional events to create a molecular clock with a period of about 24 h. The intrinsic clock is coupled to cell functions by the cyclic activation of molecular effectors which translate time keeping into changes of cell state. The mechanistic/mammalian Target of Rapamycin protein (mTOR) is a crucial effector as the periodic activation of this pathway directly couples the circadian clock to cell proteostasis and function. Importantly, mTOR plays a fundamental role in synaptic plasticity as it is implicated in the conversion of short term to long term plasticity by controlling protein synthesis in the postsynaptic volume. In this short review we will discuss the basic elements of these signaling modules and we will underline some open issues on our understanding of the complex link between circadian clock, mTOR activation and neuronal excitability.

## The cell autonomous molecular clock

**FIGURE 1:**
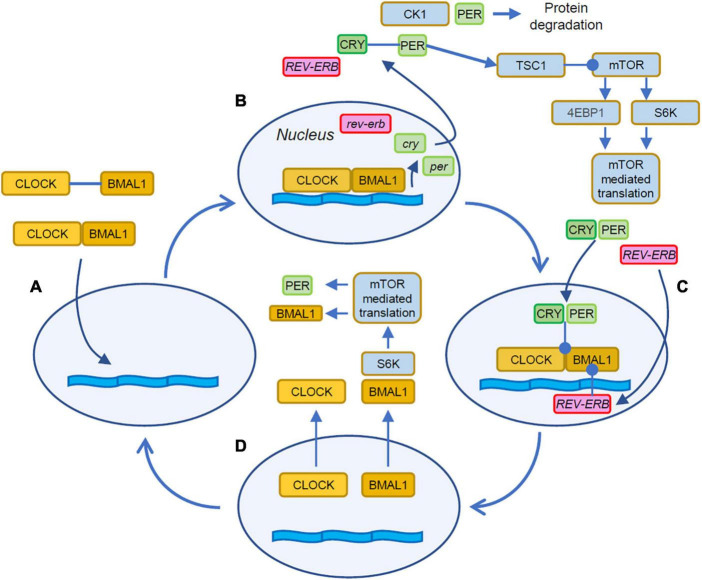
Stages of the autonomous circadian clock. **(A)** The cycle begins with the dimerization of CLOCK and BMAL1 in the cytosol and the following translocation to the nucleus. **(B)** The CLOCK:BMAL1 heterodimers bind to E-box response elements to promote the expression of several genes including *Cry* and *Per*. Cytosolic PER takes part in a regulatory loop with mTOR by activating TSC1, and upstream inhibitor of mTOR thus contributing to the entrainment of mTOR to the circadian cycle (Wu et al., 2019). PER activity is curtailed by its degradation mediated by CK1. **(C)** The CRY:PER heterodimers translocate in the nucleus and inhibit the association of CLOCK:BMAL1 with the DNA thus repressing gene expression. **(D)** BMAL1 shuttles between the nucleus and the cytoplasm where it is phosphorylated by S6K and then participates to the rhythmic activation of translation (Lipton et al., 2015). Orange boxes indicate the activator elements of the translation feedback, while elements in green and magenta indicate the repressors. Filled arrowheads indicate protein translocations and positive interactions. Lines terminating with a circle indicate inhibitory interactions.

The flow of time in cells is kept by two interacting processes that include delay and feedback lines. In most cells, the molecular clock is due to a feedback loop based on transcription and translation of promoters and repressors of transcription as illustrated in Figure 1 (Dunlap, 1999; Shearman et al., 2000; Ko and Takahashi, 2006). The molecular counterpart of the delay line is implemented by the intrinsic timing of the transcription and translation steps involved in the process and in the characteristic timing of the trafficking of the elements between nucleus and cytoplasm (Tamaru et al., 2003; Kwon et al., 2006). The description of the loop can be conveniently started with the heterodimerization of the proteins CLOCK (Circadian Locomotor Output Cycle Kaput) and BMAL1 (Brain and Muscle ARNT-Like protein 1) in the cytosol of the cell (Figure 1A). These heterodimers then translocate to the nucleus where, by binding to DNA regulatory elements, activate the expression of the *per* (Period) and *cry* (Cryptochrome, Figure 1B) genes (Darlington et al., 1998). The newly translated cytosolic PER and CRY proteins form heterodimers that eventually translocate into the nucleus (Tamaru et al., 2003; Kwon et al., 2006) where they act on the CLOCK:BMAL1 complex to repress their own transcription (Figure 1C; Gekakis et al., 1998; Sangoram et al., 1998).

On top of this feedback loop, sits a second modulatory element: the CLOCK:BMAL1 heterodimers activate the transcription of two additional genes: Rev-Erb and Ror, two retinoic acid-related orphan nuclear receptors. These two additional elements exert opposite actions on BMAL1: while REV-ERB inhibits Bmal1 transcription, ROR promotes it, leading to a complex modulation of the circadian regulation of BMAL1 abundance (Guillaumond et al., 2005; Takahashi, 2017; Cox and Takahashi, 2019). These autoregulated feedback loops complete a cycle in about 24 h and represent the circadian clock.

The period of the circadian clock also depends on the rate of degradation of its elements. Notably, Casein Kinase 1 (CK1) is a small family of kinases that targets PER to promote proteasomal degradation. The inhibition of CK1 causes a reduced rate of degradation and causes a lengthening of the circadian period (Hirota et al., 2010; Meng et al., 2010; Kolarski et al., 2021). In addition, post-translational modifications of the core elements of the circadian clock have a subsidiary role in stabilizing and modulating its operation (Mehra et al., 2009; Brenna and Albrecht, 2020). Mutations that affect phosphorylation sites of some of the core clock elements lead to a surprising variety of downstream effects. Mutated Per2 is associated to altered wake-rest cycle and sleep disturbance (Toh et al., 2001; Xu et al., 2007) due to defective degradation mediated by CK1 (Xu et al., 2005); mutations of Per1 instead leads to feeding disorder and obesity (Liu et al., 2014) and to worsened outcome of strokes (He et al., 2022). At the low end of this spectrum of effects, the defective phosphorylation of Cry1 has a limited impact on voluntary locomotion (Vaughan et al., 2019).

Finally, to underline the robustness of this machinery and its importance for cell function, there are also multiple levels of redundancy that prevent the complete failure of time keeping in case of the loss of some elements. The redundancy is provided by paralogs of the essential molecular players: CLOCK has a paralog in NPAS2 (DeBruyne et al., 2007) that can bind with BMAL1. Although the two paralogs PER1 and PER2 are not functionally identical and support different downstream functions (Park et al., 2023), they still offer a degree of redundancy since the double knock out is necessary to remove periodicity of the clock (Zheng et al., 2001). Similarly, the KO of both paralogs CRY1 and CRY2, is required for a completely arrhythmic phenotype (Vitaterna et al., 1999).

## mTOR is a bidirectional linker between the circadian clock, cell response, and environmental signals

The interaction of the autonomous circadian clock with the cell and the complete organism requires two additional components. First, the phase of the autonomous clock must be converted in biochemical actions that can be interpreted by the cell and drive its response. Second, the circadian clock must be able to receive signals from the environment to keep the alignment between the internal state and the shifting environmental conditions. The periodic transcription-translation cycle driven by the CLOCK:BMAL1 complex not only seeds the circadian clock, but also entrains several molecular effectors to the circadian cycle by controlling the transcription of a plethora of genes (Zhang et al., 2014). One of these genes that has a crucial role in the transduction of the cellular response to the circadian rhythm is mTOR (Laplante and Sabatini, 2013; Lipton and Sahin, 2014; Saxton and Sabatini, 2017). This protein kinase, conserved from yeast to mammals, regulates numerous signaling pathways which coordinate cell metabolism and growth with the environmental conditions and are fundamental for maintaining cell and organism physiology in a constantly changing internal state and external environment. mTOR is a serine/threonine protein kinase which forms the catalytic core of two complexes: mTORC1 and mTORC2 (Laplante and Sabatini, 2013; Lipton and Sahin, 2014; Saxton and Sabatini, 2017; Switon et al., 2017). The main function of mTORC1 is to control cell growth and metabolism by the activation of several anabolic pathways including protein, lipid, and nucleotide synthesis and the regulation of glucose metabolism. To favor cell growth mTORC1 also inhibits catabolic cellular processes. mTORC2 is mostly involved in cells proliferation and survival. To this end mTORC2 has a key role in the regulation of the actin cytoskeleton in addition to a variety of other functions (Saxton and Sabatini, 2017). mTOR signaling exerts a key role in the maintenance of brain physiology, being involved in several essential neuronal processes and functions, such as neuronal stem cell proliferation, neuronal circuits formation (Yoon et al., 2009; Nie et al., 2010) and maintenance (Raab-Graham et al., 2006; Jung et al., 2014), experience dependent synaptic plasticity, learning and memory (Casadio et al., 1999; Hoeffer et al., 2008; Hoeffer and Klann, 2010). mTOR is also implicated in the regulation of complex behaviors such as sleep, feeding and circadian rhythms (Cao and Obrietan, 2010). The centrality of this pathway in the brain is underlined by the fact that mTOR dysregulations are implicated in cognitive deficits, defective synaptic plasticity and epilepsy (Buffington et al., 2014; Borrie et al., 2017; Trovato et al., 2020; Nguyen and Bordey, 2021; Girodengo et al., 2022; Singla et al., 2022a).

The interplay of the circadian machinery with mTOR is still a matter of active investigation and involve the interaction of mTOR with elements of the cell autonomous clock. Translation is temporally shaped by the circadian regulation of mTOR expression (Zhang et al., 2014) and ribosome biogenesis (Jouffe et al., 2013). Further modulatory inputs to mTOR are due to by direct interactions of elements of the circadian clock to the mTOR pathway. PER and BMAL1 are involved in a complex regulatory loop with mTOR (Figures 1B, D) that is not yet completely understood (Singla et al., 2022a). The hyperactivation of mTOR due to loss of Tuberous Sclerosis Complex 1/2 (TSC1, TSC2), two upstream inhibitors of mTOR, disrupts the circadian clock and causes abnormally high levels of BMAL1 and increased expression of Per1 and Per2 (Lipton et al., 2017). On the other end, BMAL1 exerts a restraining effect on mTOR, since the genetic reduction of BMAL1 levels leads to mTOR hyperactivation (Khapre et al., 2014; Singla et al., 2022b). Furthermore, a positive regulatory contribution from the clock on mTOR-mediated translation is provided by the phosphorylation of cytosolic BMAL1 by the mTOR effector S6K (Lipton et al., 2015) leading to facilitated translation. This aligns mTOR activity to the late phase of the BMAL1 cycle when its localization is mostly cytosolic (Figure 1D). A negative feedback is provided by PER2 that has been shown to restrain TORC1 by activating TSC1 (Wu et al., 2019).

## The suprachiasmatic nucleus

**FIGURE 2:**
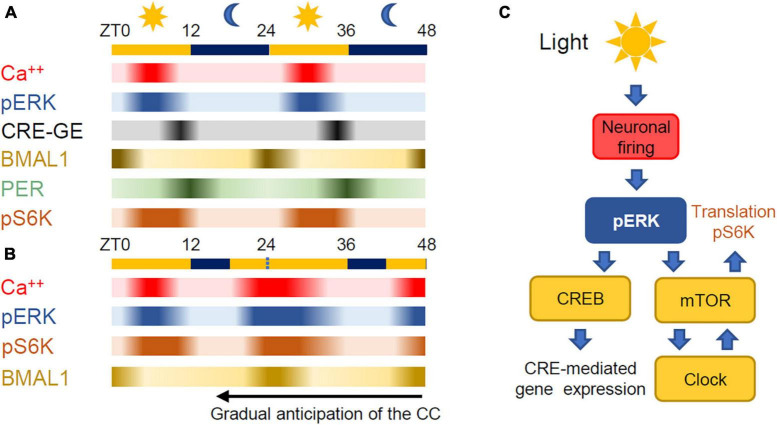
**(A)** Relative time course of the processes controlling time keeping in the SCN. ZT stands for Zeitgeber Time with 0 at dawn. Exposure to blue light at the end of darkness causes an increase of neuronal activity in the SCN and phosphorylation of ERK. The activation of the ERK pathway is followed by the activation of gene expression mediated by CREB and the activation of mTOR leading to protein synthesis. Phosphorilated S6 kinase (pS6K) is a proxy for mTORC1 activation. The temporal flow of the autonomous circadian clock is reported by the expressions of BMAL1 and PER that have a phase difference of about 12 h. **(B)** Light delivered during the late night increases firing in the SCN thus anticipating ERK phosphorylation, mTOR activation and protein synthesis. In this way, BMAL1 expression is anticipated thus leading to an overall anticipation of the circadian loop. **(C)** mTOR is the converging point between the environmental stimuli and the autonomous circadian clock.

In most metazoans, the main environmental clue is provided by the light cycle. The intersection between the diurnal light variation and the circadian machinery occurs in the suprachiasmatic nucleus (SCN) (Hastings et al., 2018; Patton and Hastings, 2018). The SCN is a small area (about 10,000–15,000 neurons in the mouse) placed in the anterior part of the hypothalamus where neurons integrate light dependent signals originating from the retina entraining the autonomous clock to the light cycle (Cao et al., 2011). The cell autonomous nature of the intrinsic clock is underlined by the fact that isolated neurons from the SCN support a diurnal change of firing rate along with changes in intracellular Ca^2+^, and exhibit a circadian expression of the gene *per2* (Webb et al., 2009; Noguchi et al., 2017), thus maintaining circadian regulation just like cultured cell lines. In the mouse SCN, the onset of neuronal firing is triggered by light exposure in the morning and leads to Extracellular Regulated Kinase (ERK) activation and to phosphorylation of CREB (Cyclic AMP Response Element Binding protein) and to the onset of CRE-mediated transcription (Ginty et al., 1993). This step is crucial for the entrainment of the circadian clock to light (Lee et al., 2010; Wheaton et al., 2018) and reflects the importance of cAMP signaling in maintenance of the circadian cycle (O’Neill et al., 2008). Following this, the levels of PER and CRY rise through the late afternoon leading to inhibition of CLOCK:BMAL1 transcription early at night. The approximate timing of all of these processes are indicated in Figure 2A (O’Neill et al., 2008; Cao et al., 2011; Brancaccio et al., 2013; Hastings et al., 2018; Nishide et al., 2018). The circadian regulation of neuronal firing in the SCN sends time keeping signals to the entire brain and body by means of synaptic transmission and hormone secretion (Herzog et al., 2017).

In the SCN, mTOR activity is regulated by the circadian clock (Cao et al., 2011) and, importantly, the relationship between mTOR activity and the circadian clock is bidirectional as changes in mTOR activity pattern driven by environmental changes can feedback to the cellular clock. Indeed, as anybody that has flown through different time zones knows, the cell autonomous clock is gradually realigned to the day-night cycle by exposure to light during the subjective dark period. The interruption of darkness in the final part of the night by an exposure to light causes a rapid build-up of ERK phosphorylation in the SCN (Obrietan et al., 1998; Serchov et al., 2016) leading to the activation of mTOR (Cao et al., 2008; Cao and Obrietan, 2010) and the anticipated expression of PER1 and PER2 (Albrecht et al., 1997; Shearman et al., 1997; Shigeyoshi et al., 1997) and BMAL1, thus anticipating the original circadian cycle (Figures 2B, C). As already mentioned, we do not have a complete picture of the interactions among the circadian clock, ERK and mTOR pathways. A quantitative understanding of these interactions requires the knowledge of the transcriptional and post-transcription mechanisms at play, the affinity and kinetics of each reaction and also the detailed description of the delays imposed by diffusion and shuttling between nucleus and cytoplasm of the involved entities (Koch et al., 2022).

The cross talk between the mTOR and ERK pathways (Switon et al., 2017) and the role of ERK in circadian rhythm biology (Obrietan et al., 1998; Akashi et al., 2008; Goldsmith and Bell-Pedersen, 2013) are remarkable as the RAS-ERK signaling axis is essential to integrate synaptic responses with the activation of tyrosine kinase receptors by permissive factors such as Brain Derived Neurotrophic Factor (BDNF) or Insulin Growth Factor (IGF), which play a crucial role in synaptic plasticity. ERK is activated by multiple pathways which can be differently recruited in different brain districts (Miningou and Blackwell, 2020). For example, as mentioned above, ERK is phosphorylated in the SCN by environmental blue light (Obrietan et al., 1998) while in the visual cortex the visual stimulus needs to be spatially patterned to activate the pathway (Cancedda et al., 2003).

In neurons, mTOR is crucial not only for cell housekeeping but also for local protein synthesis in dendrites, in dendritic spines and in axons (Altas et al., 2022). Indeed, activity dependent local activation of mTOR is required for consolidation of long-term memory (Nguyen, 2002; Cammalleri et al., 2003; Kelleher et al., 2004; Tsokas et al., 2007; Buffington et al., 2014). In large pyramidal neurons, the biochemical coupling between soma, dendrites and dendritic spines is only partial (Logan and McClung, 2019; Walker et al., 2020). Thus, in our view, an important open question about the cross talk between circadian clock and synaptic plasticity is whether there are two pools of mTOR in neurons. First, a perisomatic pool tightly associated with the circadian time keeping and responsible for homeostasis of the neuronal proteasome. Second, a dendritic pool, which activity is less strongly coupled to the cell clock and that is mostly modulated by the history of local synaptic activity and by the availability of ligands of the tyrosine kinase receptors.

## Circadian clock, synaptic plasticity, and cortical excitability

The importance of circadian rhythm is underlined by the link between circadian clock and proper brain operations, as disruption of the circadian rhythm prevents normal cognitive functions and can result in brain pathology (Logan and McClung, 2019; Walker et al., 2020). Everybody knows that the time of the day influences cognitive performances. The link between circadian rhythm and behavioral performances has been explored first in a mouse model in a seminal paper of several decades ago (Davies et al., 1973). In that study rats were allowed to explore an illuminated environment joined to a smaller dark chamber where they were subjected to electric shock. After 24 h rats were placed again in the experimental environment and the degree of passive avoidance was measured as the amount of time that the rat preferred to spend in the illuminated chamber. The avoidance response was much larger when training and testing were performed during the light phase. The authors concluded that this result *“probably reflects a 24-h rhythm in some aspects of learning or retention.”* Other studies in nocturnal rodents confirm that learning assays involving negative stimuli peaks during the day (Chaudhury and Colwell, 2002; Eckel-Mahan et al., 2008; Wang et al., 2009), thus suggesting a increased plasticity of the pathways processing negative emotions. In contrast, behavioral assays of memory and learning involving positive or neutral stimuli peaked at night (reviewed in Snider et al., 2018; Goode et al., 2022). From this body of evidence, we can expect that long-term potentiation (LTP), a correlate to memory and learning, should also be modulated through the day-night cycle. Indeed, studies performed in nocturnal rodents show that hippocampal LTP is remarkably affected by the time of the day and that this modulation stands even after rearing the animals in darkness for a few days, thus demonstrating the dependency on the intrinsic circadian clock rather than on the light cycle. Surprisingly, there is no complete agreement on the direction of the effect: while several studies indicate that hippocampal LTP measured in CA1 is stronger at night, during the active phase of nocturnal animals (Chaudhury et al., 2005; Bowden et al., 2012; Nakatsuka and Natsume, 2014; Besing et al., 2017; Davis et al., 2020; Goode et al., 2022), other studies provide a different picture, with the peak of LTP during the day (Raghavan et al., 1999; McCauley et al., 2020). These differences underline the complexity of the problem of linking circadian rhythm with models of synaptic plasticity and behavioral assays. Notwithstanding these differences, that might arise from diverse factors such as animals age, timing of the slice preparation and other experimental details, the link between LTP, time of day and circadian clock is unquestionable.

This connection is strengthened by the observation that genetic alterations of the circadian clock lead to memory and LTP impairment. The ablation of the *per1* (Rawashdeh et al., 2014) or *bmal1* (Wardlaw et al., 2014) genes in mice impairs not only a proper operation of the circadian clock, but it also degrades memory and hippocampal LTP. Finally, in the APP/PS1 mouse model of Alzheimer’s disease, the impairment of circadian rhythm is accompanied by loss of the circadian difference for novel object recognition and hippocampal LTP (He et al., 2020).

Given the bidirectional involvement of mTOR and RAS-ERK pathways as both entrainers and effectors of the circadian rhythm and the role that these pathways play in synaptic plasticity it is not surprising that there is a tight link between plasticity and the circadian clock as supported by a wide body of literature (Saraf et al., 2014; Snider et al., 2018). Here, we would like to emphasize very recent data that suggest the relevance of a novel mechanism, so far only identified in non-excitable cells and cardiomyocytes, coupling circadian activation of mTOR with intracellular ion homeostasis. This mechanism is suggested by a manifestation of circadian biology that has been known for several years but it has not permeated the neurobiology community yet: during the circadian cycle the intracellular concentration of Na^+^, K^+^, Cl^–^, and Mg^2+^ oscillates in phase (Feeney et al., 2016; O’Neill et al., 2020). This process was demonstrated first in non-excitable cells, until a recent beautiful study has demonstrated that the circadian clock causes a periodic change of intracellular K^+^, Na^+^ and Cl^–^ in cardiomyocytes (Stangherlin et al., 2021). As expected, this process influences cells excitability leading to a diurnal cycle of the frequency of the spontaneous heartbeat that accelerates in correspondence of elevated intracellular Na^+^ and K^+^. The periodic changes in intracellular ion concentration are controlled by ion cotransporters and this is driven by the cyclic circadian activation of mTOR so that high ion concentration occurs in correspondence of low mTOR activity (Stangherlin et al., 2021). Importantly, flattening mTOR activity by its pharmacological inhibition cancels the circadian rhythm of heartbeat frequency.

If changes of intracellular Na^+^ and K^+^ occurred also in neurons, they would affect the resting membrane potential during the circadian cycle and the amplitude of action potentials. Since electroneutrality must be maintained, the circadian influx or efflux of Na^+^ and K^+^ must be compensated by a correspondent flux of Cl^–^. The presence of a circadian oscillation of intracellular Cl^–^, would have complex effects on neuronal excitability mediated by changes of the driving force of the current flowing through the ionotropic GABA_A_ receptor. This is the situation present in neurons of the SCN where intracellular Cl^–^ changes under the control of the circadian clock with the peak concentration reached during the day, and the nadir at night (Wagner et al., 1997). This rhythmicity is maintained even in isolated neurons from the SCN (Shimura et al., 2002). Intracellular Cl^–^ influences the size of GABA_A_ currents, and it can be considered as a master regulator of neuronal inhibition. In the SCN the circadian change of intracellular Cl^–^ modulates the postsynaptic effects of GABA and contributes to the control of the periodic activity of this network (McNeill et al., 2018; Ono et al., 2018).

Up to now, it has been assumed that the modulation of baseline intracellular Cl^–^ during the day was a peculiarity of the SCN. However, two very recent studies suggest that a diurnal change of Cl^–^ might be a more general feature of cortical physiology. In the first study intracellular Cl^–^ has been estimated in slices by means of the measurement of the reversal of the GABA_A_ currents. It was observed that intracellular Cl^–^ increased at night during the active phase and the authors hypothesized that the Cl^–^ load encodes for sleep pressure (Alfonsa et al., 2023). The second study employed a genetically encoded sensor for intracellular Cl^–^ and two photon *in vivo* imaging (Lodovichi et al., 2022) to detect a large physiological diurnal fluctuation of baseline Cl^–^ inside cortical pyramidal cells, with high Cl^–^ when mice are awake (night), relative to when they are usually asleep (day) (Pracucci et al., 2022). This concentration shift causes a drastic increase of neuronal excitability at night as witnessed by a lowered epileptic threshold. The increased excitability at night can be countered by bumetanide, an inhibitor of the cotransporter NKCC1 that import Cl^–^ in neurons. Although neither studies demonstrated that the observed diurnal change of intracellular Cl^–^ is directly linked to the circadian clock, it is tempting to speculate that the observed changes are, at least to a certain extent, due to the same mechanisms observed in cardiomyocytes (Stangherlin et al., 2021). These data might contribute to explain the dependency of LTP on the time of the day. If the intracellular chloride of CA1 pyramidal neurons was higher at night, this would attenuate the inhibitory currents compared to daytime thus leading to enhanced LTP. This interpretation is consistent with the fact that the moderate LTP observed by day can be increased to the level reached at night by Gabazine, an antagonist of GABA_A_ receptors (Nakatsuka and Natsume, 2014). More in general several mechanisms have been implicated in circadian changes of neuronal excitability (Paul et al., 2020) and these changes are likely to affect the early phase of LTP. Furthermore, consolidation of LTP, that is well known to depend on translation and transcription in the post synaptic domain, can also be modulated by the circadian clock via mTOR (Kelleher et al., 2004; Hoeffer and Klann, 2010). However, we should be cognizant of the fact that we ignore if the rules of circadian activity of mTOR also extend to the post synaptic volume. Another important signaling axis that has recently been implicated in circadian regulation of memory and learning is the MNK-eIF4E (mitogen activated protein kinase-interacting kinase-eukaryotic translation initiation factor). This pathway is required for hippocampal LTP consolidation (Hoeffer et al., 2013) and it has been shown that MNK-mediate phosphorylation of eIF4E is regulated along a daily cycle with a peak during the day and its activity modulates novel object recognition (Liu et al., 2022). Finally, it should be noted that astrocytes play a crucial role in the circadian activity of the SCN (Hastings et al., 2022) and, although we know very little about their circadian biology in the cortex, it has been recently demonstrated their participation in the circadian modulation of plasticity in the hippocampus (McCauley et al., 2020).

## Circadian rhythm, sleep, and plasticity

The alternation of sleep and wakefulness is the most obvious behavioral correlate of circadian rhythm and, given the role of sleep on brain plasticity, memory formation and consolidation (Smith, 1996; Smith and Rose, 1996; Stickgold, 1998; Tononi and Cirelli, 2014; Findlay et al., 2020) and this is an important area of intersection between circadian mechanisms and synaptic plasticity. In rodents, memory storage for several different tasks is impaired when REM sleep deprivation follows the training-learning phase (Fishbein, 1971; Zamore and Veasey, 2022). The seminal study by Wilson and McNaughton demonstrated that during slow wave sleep (and during period of inactivity) hippocampal neurons fire intermittent, synchronized bursts (ripples) that replay the pattern of activity representing the behavior performed in the prior awaking period (Wilson and McNaughton, 1994). In the following years, the role of sharp wave ripples in memory consolidation has been confirmed by studies performed in behaving animals, *in vitro* human tissue and by modeling studies (Buzsáki and da Silva, 2012; Joo and Frank, 2018). Synapses cannot follow a trajectory in which they are only strengthened by experience and not all the information and tasks experienced during the awake period are worthy to be consolidated and preserved. Thus, synapses must undergo downscaling during sleep, to remove irrelevant synapses and to allow the incorporation of new information in the next awake cycle in the global synaptic pool. To seek to understand the mechanism underlying synaptic homeostasis and how the sleep-awake cycle contribute to it, Tononi and Cirelli (2014) proposed an elegant idea, the synaptic homeostasis hypothesis (SHY), according to which during the awake phase, learning about relevant environmental stimuli results in strengthening of synapses throughout the brain. During sleep, spontaneous activity weakens synaptic connections and repristinates synaptic homeostasis (Tononi and Cirelli, 2014). Overall, the picture that emerges is that sleep operates both the consolidation of salient memory and enables weakening of previously strengthened synapses to restore homeostasis and enable another cycle of learning and memory. Thus, even this simplified analysis shows that the two phases of the learning process, namely, the experience dependent plasticity and the homeostatic rearrangement of synapses, are roughly distributed at two opposite poles of the circadian cycle.

Synaptic strength and function in wake and sleep are coupled to distinct genetic signatures. In rodents, several microarray experiments indicate that wakefulness is associated to the expression of activity-regulated genes which control experience dependent plasticity. The expression of these genes (such as *Arc*, *Bdnf*, *Homer1a*) was found to be modulated by ERK. In rodents, ERK phosphorylation in cortical neurons increases or decreases with wake and sleep. Indeed, deletion or inhibition of ERK phosphorylation were shown to modulate wake and sleep duration (Mikhail et al., 2017). Therefore, the circadian regulation of ERK signaling pathway correlates the waking experience with synaptic plasticity, while sleep renormalizes synaptic strength, in line with the hypothesis that *“sleep is the price for synaptic plasticity”* (Tononi and Cirelli, 2014). The cyclic activation of mTOR, that was shown to contribute to memory consolidation in hippocampus (Saraf et al., 2014; Snider et al., 2018) is likely to contribute to this more general process of synaptic homeostasis, although direct evidence is still missing. Circadian oscillations of the ERK signaling pathway were reported to regulate complex functions such as sleep and learning and memory also in invertebrate, highlighting the robustness of such circadian intracellular signaling cascade in regulating synaptic homeostasis from fly to mammals. Sleep deprivation and social enrichment was found to increase ERK phosphorylation, while disruption of ERK signaling pathway reduced sleep duration and prevented neuronal plasticity triggered by social environmental enrichment. Using a CRE luciferase reporter in flies, ERK phosphorylation was shown to be coupled to CREB activation since CRE-luciferase activity increased with ERK phosphorylation and was reduced by ERK disruption (Vanderheyden et al., 2013). These data again indicate that the diurnal regulation of ERK signaling translates neuronal activity in synaptic plasticity and controls sleep and wake duration. This regulation is biunivocal, as disrupting sleep/wakefulness duration alters ERK signaling and neuronal plasticity.

The awake-sleep cycle appears important also for the full expression of plasticity during the critical period, when neuronal activity shapes the growing circuits by weakening and strengthening specific synapses to sculpt the mature architecture of neuronal connections. Previous studies showed that ocular dominance plasticity is triggered by monocular deprivation during wakefulness and consolidated during the following sleep (Frank et al., 2001; Dumoulin et al., 2015). How sleep could consolidate ocular dominance plasticity remained obscure. ERK signaling pathway was shown to be critical for experience dependent plasticity in the visual cortex as inhibition of ERK pathway prevents cortical LTP and the shift of ocular dominance toward the open eye (Di Cristo et al., 2001), furthermore, ERK activation during sleep is required for ocular dependent plasticity consolidation (Dumoulin et al., 2015). Although the mechanism underling ERK dependent consolidation is still unclear, it is likely that this signaling pathway modulates the expression of genes involved in synaptic plasticity such as *bdnf*. Deepening the understanding of the role of sleep on synaptic homeostasis, it was found that following monocular deprivation in rodents, REM sleep promotes synaptic strengthening or weakening in different cortical layers (Renouard et al., 2022). These data indicate that the effects of sleep on synaptic plasticity are circuit specific and depend on the previous waking experience. The molecular basis of this process remains elusive. It is worth noticing that sleep modulates experience dependent plasticity, but it is also affected by waking experience. Slow wave activity during sleep is significantly reduced upon dark rearing, during the critical period in cats and rats (Miyamoto et al., 2003). All together these results suggest that sleep and ERK signaling pathway associated to sleep-wake cycle are critical for normal brain development. Indeed, most neurodevelopmental disorders, in particular autism spectrum disorders (ASD), are associated to sleep disturbances. The molecular basis contributing to the pathogenesis of these diseases is mostly related to alterations in proteins associated to synaptic plasticity, that as we have seen in this brief review, are critically regulated by the wake-sleep cycle through the phasic activation of ERK and mTOR pathways (Medina et al., 2023).

The existence of a diurnal rhythm of intracellular Cl^–^ in the brain (Pracucci et al., 2022) and its bidirectional relationship with sleep (Alfonsa et al., 2023) might represent an important connection between circadian clock, neuronal excitability and brain homeostasis.

## Conclusion

Work and societal habits are increasing the separation between the periodic need for rest from the natural day-light cycle. Furthermore, physiological rhythms can also be disrupted by life habits: since the circadian clock is exquisitely sensitive to the exposure to blue light during the rest phase, the widespread use of electronic devices at night subtly interferes with the correct cycle maintenance. Disturbances of the natural circadian rhythm invariably leads to negative consequences, including sleep disturbances, attention deficits with consequent societal challenges due to loss of performances, and increased susceptibility to mistakes and accidents. Besides the more apparent consequences on health and wellbeing, the disruption of the circadian rhythm has widespread consequences on cognition, memory and learning. We suggest that shedding light on the relationship between circadian biology, neuronal excitability and synaptic plasticity will lead to a better understanding of these key aspects of brain function that are rooted in a fundamental feature of biological systems.

## Author contributions

CL and GR wrote this review. Both authors contributed to the article and approved the submitted version.
